# Bacteriophages as post-harvest biocontrol agents against *Campylobacter* in food matrices: a systematic review of efficacy, taxonomic diversity, and regulatory perspectives

**DOI:** 10.3389/fnut.2026.1897467

**Published:** 2026-07-30

**Authors:** Sarah Ramalho Monteiro Gomes, Hannay Crystynah Almeida de Souza, Ana Beatriz Portes, Carlos Adam Conte-Junior, Pedro Panzenhagen

**Affiliations:** 1 Center for Food Analysis (NAL), Technological Development Support Laboratory (LADETEC), Institute of Chemistry (IQ), Federal University of Rio de Janeiro (UFRJ), Rio de Janeiro, Brazil; 2 Department of Biochemistry, Laboratory of Advanced Analysis in Biochemistry and Molecular Biology (LAABBM), Institute of Chemistry (IQ), Federal University of Rio de Janeiro (UFRJ), Rio de Janeiro, Brazil; 3 Analytical and Molecular Laboratory Center (CLAn), Institute of Chemistry (IQ), Federal University of Rio de Janeiro (UFRJ), Rio de Janeiro, Brazil; 4 Graduate Program in Food Science (PPGCAL), Institute of Chemistry (IQ), Federal University of Rio de Janeiro (UFRJ), Rio de Janeiro, Brazil; 5 Graduate Program in Biochemistry (PPGBq), Institute of Chemistry (IQ), Federal University of Rio de Janeiro (UFRJ), Rio de Janeiro, Brazil; 6 Department of General Microbiology, Graduate Program in Microbiology (PPGMicro), Institute of Microbiology Paulo de Góes (IMPG), Federal University of Rio de Janeiro (UFRJ), Rio de Janeiro, Brazil

**Keywords:** bacteriophages, biological control agents, *Campylobacter coli*, *Campylobacter jejuni*, food microbiology, food safety, phages

## Abstract

*Campylobacter* is the leading cause of bacterial foodborne gastroenteritis globally, with poultry meat as the primary transmission vehicle representing a persistent public health challenge. Following PRISMA 2020 guidelines, a systematic search of PubMed, Embase, and Web of Science comprised 14 eligible original studies evaluating lytic bacteriophage application against *Campylobacter* in post-harvest food matrices. Statistically significant bacterial reductions were reported in 12 of 14 studies (*p* < 0.05), ranging from 0.2 to ≥5 log_10_ CFU/g, with efficacy strongly modulated by temperature, MOI, and matrix composition. Under refrigeration conditions, reductions were generally modest (0.5–1.5 log_10_), whereas synergistic physical-biological combinations consistently exceeded the 2 log_10_ EFSA-referenced risk-reduction threshold. Group II (*Firehammervirus*) and Group III (*Fletchervirus*) phages exhibited complementary host-range and thermal profiles, providing the mechanistic rationale for multi-group cocktail formulations. Genomic screening of all characterized candidates confirmed the absence of lysogeny, virulence, and antimicrobial resistance determinants. These findings demonstrate that lytic bacteriophages constitute a biologically efficient and genomically safe post-harvest biocontrol strategy, whose industrial translation can optimize high-MOI formulations, multi-group cocktails, and regulatory frameworks validated under commercially relevant conditions.

## 1 Introduction

*Campylobacter*, mainly *Campylobacter jejuni* (*C. jejuni*) and *Campylobacter coli* (*C. coli*), is recognized globally as the leading cause of bacterial gastroenteritis in humans. It is one of the four main global causes of diarrheal diseases, which are the most common illnesses resulting from unsafe food, affecting 550 million people yearly (1). Although infections are often self-limiting, campylobacteriosis may lead to severe post-infection complications, including Guillain-Barré syndrome, reactive arthritis, and irritable bowel syndrome (2). In addition to its clinical impact, campylobacteriosis imposes a substantial socioeconomic burden. In the European Union, the disease burden of campylobacteriosis and its sequelae has been estimated at 0.35 million disability-adjusted life years (DALYs) per year, with total annual costs of approximately €2.4 billion (3). In the United States, the annual economic burden of foodborne *Campylobacter* spp. has been estimated at US$2.18 billion in 2018 dollars, including medical costs, productivity losses, and disease-associated mortality (4).

Warm-blooded animals are commonly colonized by *Campylobacter*, with poultry representing the main source of human infection (5). In broiler chickens, *Campylobacter* asymptomatically colonizes the intestinal tract and can reach concentrations of up to 8 log_10_ CFU/g in the cecum (6), while in humans, approximately 500–800 CFU are sufficient to cause infection (7) making *Campylobacter* a major food safety concern. Considering the public health impact of this pathogen, the EU has established a maximum limit of 1,000 CFU/g in chicken carcasses after chilling as part of a microbiological risk-reduction strategy in the poultry production chain (8). Consequently, it turns essential the implementation of effective measures to minimize *Campylobacter* contamination throughout poultry production.

Several strategies have been employed throughout the poultry production chain to minimize *Campylobacter* contamination, including biosecurity measures, cleaning and disinfection practices, as well as the use of prebiotics and probiotics (9). Despite being considered a fastidious microorganism under *in vitro* culture conditions, *Campylobacter* possesses adaptive and survival mechanisms that enable its persistence throughout the meat production chain, even under good sanitary practices (2, 6). Once a poultry flock is exposed to the pathogen, controlling its dissemination within broiler farms becomes extremely difficult because of its rapid spread of colonization (10, 11). During slaughter, particularly at the evisceration stage, carcass contamination may occur, representing a significant risk for human infection.

In this context, bacteriophages have emerged as a promising and environmentally sustainable complementary approach for *Campylobacter* control, acting as biocontrol agents capable of specifically lysing the pathogen (5, 12). Due to their narrow host range, bacteriophages can target specific bacteria without substantially affecting the intestinal microbiota. In addition, lytic bacteriophages are generally considered safe for food applications due to the absence of virulence and antimicrobial resistance genes, while also preserving the sensory properties of food products (2, 6). Therefore, this systematic review aimed to (i) evaluate the efficacy of lytic bacteriophages as post-harvest biocontrol agents against *Campylobacter* spp. in food matrices; (ii) characterize the taxonomic diversity and receptor specificity of therapeutic phage candidates; (iii) assess the influence of physicochemical parameters and food matrix characteristics on phage efficacy; and (iv) examine the regulatory landscape and safety profiles of phage-based interventions in food systems.

## 2 Materials and methods

### 2.1 Systematic review criteria

To synthesize current scientific evidence regarding the efficacy of lytic bacteriophages as a biocontrol strategy against *Campylobacter* spp. in food matrices, a comprehensive electronic database search was conducted on March 12, 2026. The search targeted three primary databases: Pubmed, Embase and Web of Science. The search terms included: (*Campylobacter* OR “*Campylobacter jejuni*” OR “*Campylobacter coli*”) AND (bacteriophage OR phage OR “phage cocktail”) AND (biocontrol OR biological control OR control OR bacterial reduction OR reduction OR inactivation OR antibacterial activity OR decontamination OR mitigation) AND (food OR poultry OR chicken OR meat OR “food matrix”). No publication date restrictions were applied, and all studies indexed in the selected databases up to March 12, 2026, were considered eligible for screening. Grey literature sources, including conference proceedings, dissertations, theses, monographs, and other non-peer-reviewed documents, were not included in the search strategy. This approach was adopted to ensure methodological consistency and the inclusion of studies subjected to formal peer-review processes. This systematic review was conducted in strict accordance with the Preferred Reporting Items for Systematic reviews and Meta-Analyses (PRISMA) guidelines (13).

Article screening and management were performed using Rayyan. The methodological workflow consisted of four stages: (i) initial import of references; (ii) identification and removal of duplicates; (iii) primary screening of titles and abstracts according to the predefined eligibility criteria; and (iv) full-text assessment to confirm final inclusion. Title/abstract screening and full-text evaluation were performed independently by two reviewers. Disagreements were initially resolved through discussion between the two reviewers, with reassessment of the full-text article when necessary. If consensus could not be reached, a third reviewer was consulted, and the final decision was made by consensus. This procedure was adopted to minimize selection bias and improve the reproducibility of the study selection process.

To validate the search and screening process, all records identified through the electronic databases were independently assessed by two reviewers. Disagreements regarding study eligibility were resolved through discussion and consultation with a third reviewer. This procedure was implemented to minimize selection bias and enhance the reliability and reproducibility of the review process.

### 2.2 Focus questions

The review’s main question was structured according to the PICO framework: Population (P) consisted of *Campylobacter* spp., particularly *Campylobacter jejuni* and *Campylobacter coli*, present in food matrices such as poultry, chicken, and meat; Intervention (I) included the application of bacteriophages or phage cocktails for bacterial control; Comparison (C) involved untreated contaminated samples or control treatments without bacteriophage application; Outcome (O) was defined as the reduction, inactivation, or decontamination of *Campylobacter* in food matrices. Based on this framework, this review addresses the following central research question: (i) Does the application of bacteriophages effectively reduce bacterial contamination in food matrices contaminated with *Campylobacter* spp.?

Furthermore, the review explores several critical secondary questions: (ii) Do MDR *Campylobacter* isolates also exhibit phage resistance?; (iii) Which phage taxonomic groups demonstrate superior bactericidal efficacy?; (iv) What target receptors were more susceptible to the phage activity?; (v) What is the correlation between the bacterial inoculum and Multiplicity of Infection (MOI) for effective reduction and biofilm development interference?; (vi) Do the food matrix characteristics and storage conditions impact phage stability and antibacterial efficacy?; (vii) What is the comparative efficacy, expressed as logarithmic reduction, between single phages and phage cocktails use?; (viii) Do phage biocontrol exhibit a synergetic or additive effect when combined with physical and chemical methods, such as UV irradiation or freezing?; (ix) How is the geographic distribution of eligible research, and which regions demonstrate the most significant advancement in *Campylobacter* phage biocontrol studies? And (x) what are the human safety and regulatory implications associated with phage-mediated biocontrol in food systems?

### 2.3 Selection criteria

#### 2.3.1 Inclusion criteria

Original and peer-reviewed studies; Articles describing the use of lytic bacteriophages for the biocontrol of *Campylobacter* spp. in food matrices; Studies from all years were included to ensure a comprehensive chronological overview.

#### 2.3.2 Exclusion criteria

Literature reviews, books, cost-utility analysis, editorials and incomplete articles; Monographs, conferences and academic theses; Other bacteria than *Campylobacter* spp.; *In vitro* and *in vivo* matrices; pre-harvest studies; Methodologies that the primary objective was not the evaluation of phage biocontrol; Studies written in a language other than English.

### 2.4 Data extraction

Data extraction was performed using a standardized spreadsheet developed specifically for this review. The extracted variables included author and year of publication, country of study, food matrix, *Campylobacter* species, bacterial strain or isolate characteristics, antimicrobial resistance profile when available, phage identity, phage taxonomic group, receptor specificity when reported, application method, bacterial inoculum, phage concentration or MOI, storage temperature, exposure time, treatment conditions, log reduction, statistical significance, and genomic safety information, including the absence of lysogeny-associated genes, virulence determinants, and antimicrobial resistance genes when available.

Data extraction was conducted by one reviewer and independently checked by a second reviewer. Any inconsistencies were resolved through discussion and verification against the original full-text articles. When disagreement persisted, a third reviewer was consulted until consensus was reached.

### 2.5 Assessment of risk of bias and study quality

Due to the methodological heterogeneity of the included studies, particularly regarding food matrices, bacterial inoculum levels, phage application protocols, MOI values, storage temperatures, exposure times, and outcome measurements, conventional risk-of-bias tools such as Cochrane or SYRCLE were not directly applicable. Therefore, a structured qualitative assessment was performed to evaluate the methodological robustness and comparability of the included studies.

The assessment considered the following domains: (i) representativeness of the food matrix and relevance to post-harvest conditions; (ii) presence of appropriate untreated or non-phage control groups; (iii) clear reporting of bacterial strain, inoculum level, phage identity, phage concentration, and MOI; (iv) description of storage conditions, temperature, exposure time, and physicochemical parameters; (v) use of biological and/or technical replication; (vi) application of statistical analysis to support efficacy claims; (vii) reporting of antibacterial efficacy as log reduction; and (viii) genomic characterization of phages regarding the absence of lysogeny-associated genes, virulence determinants, and antimicrobial resistance genes.

This assessment was not used as an exclusion criterion but rather to contextualize the strength and limitations of the available evidence. The main potential sources of bias identified included the predominance of poultry-based matrices, limited evaluation of red meat and other food matrices, heterogeneity in phage delivery methods, inconsistent reporting of MOI and replication, and limited standardization of efficacy endpoints across studies.

## 3 Results

### 3.1 Systematic literature search

**Figure 1:**
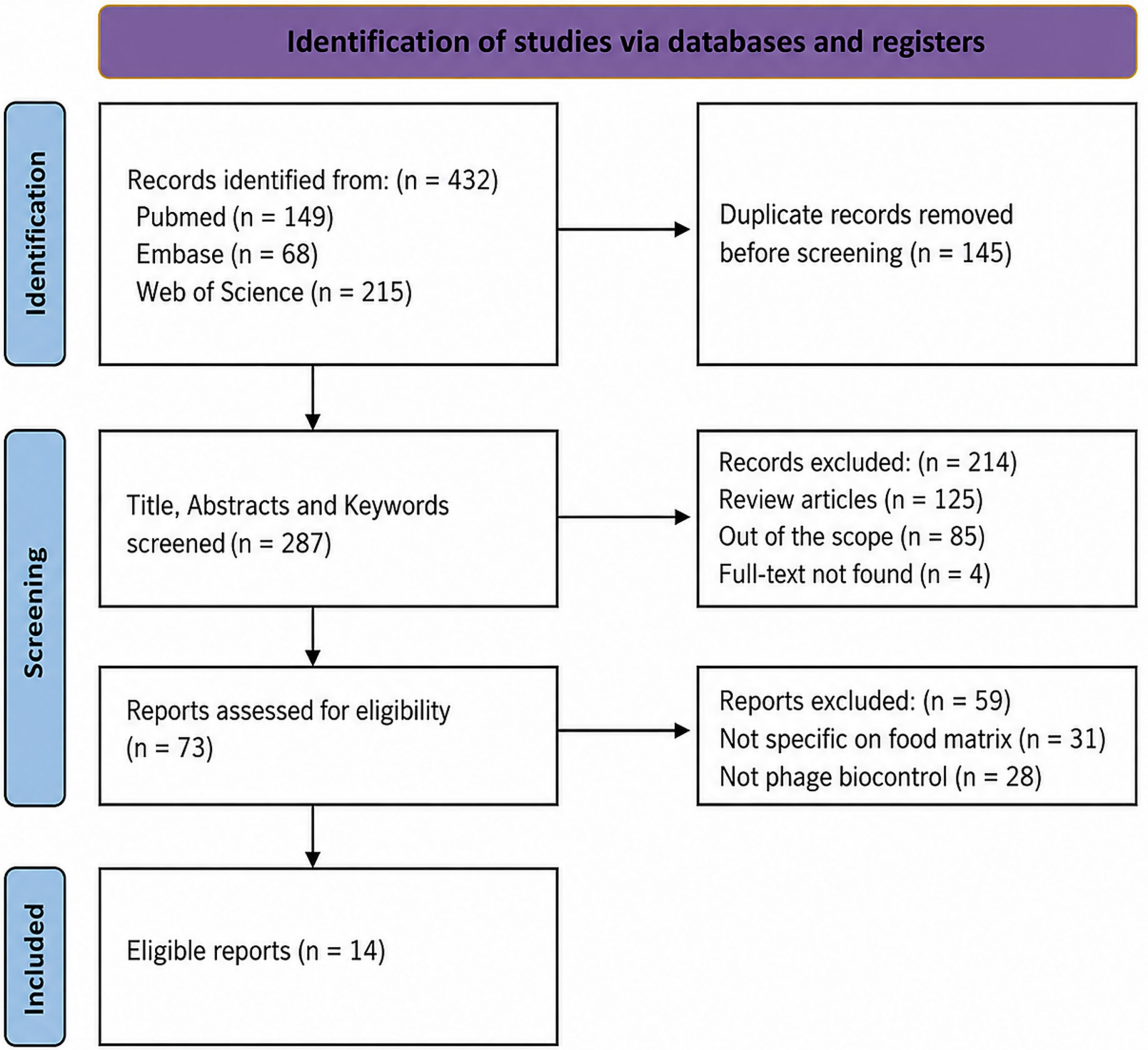
PRISMA 2020 flow diagram for new systematic reviews which included searches of databases and registers only.

The systematic literature search retrieved a total of 432 records across the three selected electronic databases: PubMed (*n* = 149), Embase (*n* = 68) and Web of Science (*n* = 215). Following rigorous deduplication based on title and authorship, 145 records were removed, leaving 287 unique records for initial title and abstract screening. Of these, 214 were excluded based on the predefined eligibility criteria, yielding 73 full-text articles for secondary assessment. After a comprehensive full-text evaluation, 59 articles were excluded for being out of scope, resulting in the final inclusion of 14 research articles for the development of this systematic review, as shown in the PRISMA flowchart (Figure 1).

### 3.2 Study selection and characteristics

**Figure 2:**
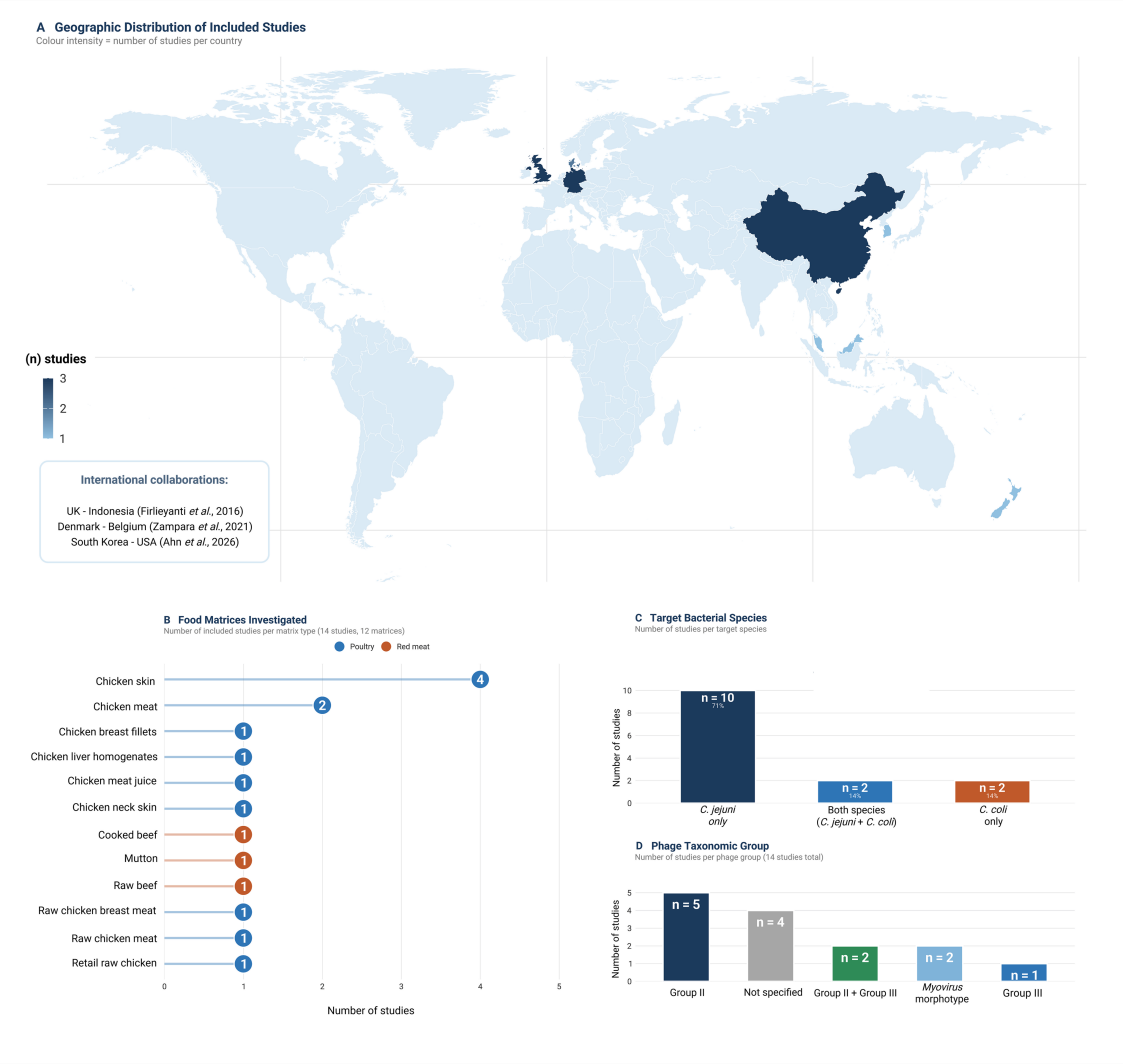
Overview of systematic review data regarding Campylobacter phage biocontrol. **(A)** Geographic Distribution of Included Studies: A world map heatmap highlighting the countries by the number of included studies, with the highest concentration in China, Germany, and the United Kingdom. A detailed legend specifies international collaborations between the United Kingdom-Indonesia, Denmark-Belgium, and South Korea-United States. **(B)** Food Matrices Investigated: A bar graph quantifying the different types of food matrices evaluated in the studies, categorized into poultry (e.g., chicken skin, meat, and liver) and red meat (e.g., beef and mutton). **(C)** Target Bacterial Species: A graph illustrating the distribution of target species, with Campylobacter jejuni being the focus of 71% of the studies, followed by studies targeting both species (C. jejuni and C. coli) or C. coli only. **(D)** Phage Taxonomic Group: A presentation of the phage taxonomic groups studied, showing the predominance of Group II (Firehammervirus), followed by unspecified groups, Group III (Fletchervirus), and Myovirus morphotypes.

The 14 included studies were published between 2003 and 2026 and delimited research from seven countries: the United Kingdom (UK) (*n* = 3), China (*n* = 3), Germany (*n* = 3), Denmark (*n* = 2), New Zealand (*n* = 1), Republic of Korea (*n* = 1), and Malaysia (*n* = 1). Three studies represented a collaboration between two different countries: a collaboration between the United Kingdom and Indonesia (14), a collaboration between Denmark and Belgium (15), and a collaboration between Republic of Korea and the United States (6) (Figure 2A).

All included studies evaluated the application of lytic bacteriophages against *Campylobacter* spp. in food matrices under post-harvest conditions. The primary food matrices investigated were chicken meat and skin (*n* = 11), followed by chicken liver homogenates (*n* = 1), chicken meat juice (*n* = 1), beef (*n* = 1), and mutton (*n* = 1) (Figure 2B). The target bacterial species were predominantly *C. jejuni* (*n* = 10), *C. coli* (*n* = 2), or a combination of both species (*n* = 2) (Figure 2C).

### 3.3 Application of bacteriophages to control the growth of *Campylobacter* spp. in food matrices

Bacteriophage application demonstrated measurable antibacterial efficacy against *Campylobacter* spp. across a diverse range of food matrices, predominantly poultry products. Of the 14 included studies, 12 reported statistically significant reductions in bacterial levels (*p* < 0.05) compared to untreated controls.

The predominant application strategy was the direct surface inoculation or spray application of phage suspensions onto chicken skin or meat. Bigwood et al. (16), was the only study to evaluate a bovine matrix, assessing phage Cj6 on both raw and cooked beef under refrigeration (5 °C) and ambient (24 °C) conditions. Thung et al. (17), was the only study to evaluate phage application by spray on both chicken meat and mutton simultaneously, using phage CJ01 stored at 4 °C, achieving reductions of 1.68 log_10_ CFU/g and 1.70 log_10_ CFU/g, respectively (*p* < 0.01). Henz et al. (18), employed a combined physical-biological approach using UV-A and UV-C irradiation in conjunction with phage C11 on chicken breast fillets, while Zampara et al. (15), applied engineered Innolysin proteins, rather than replicating phages, directly onto chicken skin under modified atmosphere packaging, achieving reductions of up to 1.63 ± 0.46 log_10_ (Cj1) and 1.18 ± 0.10 log_10_ (Cj5).

The application of individual phages was far more common (*n* = 12) than phage cocktails (*n* = 1) (19). The cocktail study demonstrated a statistically significant synergistic effect (F356: *p* = 0.02; F357: *p* = 0.03), yielding a composite reduction of 0.73 log_10_ CFU/g compared with non-significant reductions by each phage applied individually. Storage temperatures ranged from −20 °C (freezing) to 42 °C (simulating poultry processing temperatures). Most experiments (12/14, 85.7%) were conducted at refrigeration temperatures (4-5 °C), with exposure times ranging from 45 min (15) to 10 days (11). Phage-derived proteins, specifically engineered Innolysins, were utilized instead of replicating phages as an innovative technology (*n* = 1), exceeding the performance of replicating phage cocktails previously tested by the same researchers under similar *in situ* conditions (15).

### 3.4 Efficacy outcomes of bacteriophage application: log reduction

**Figure 3:**
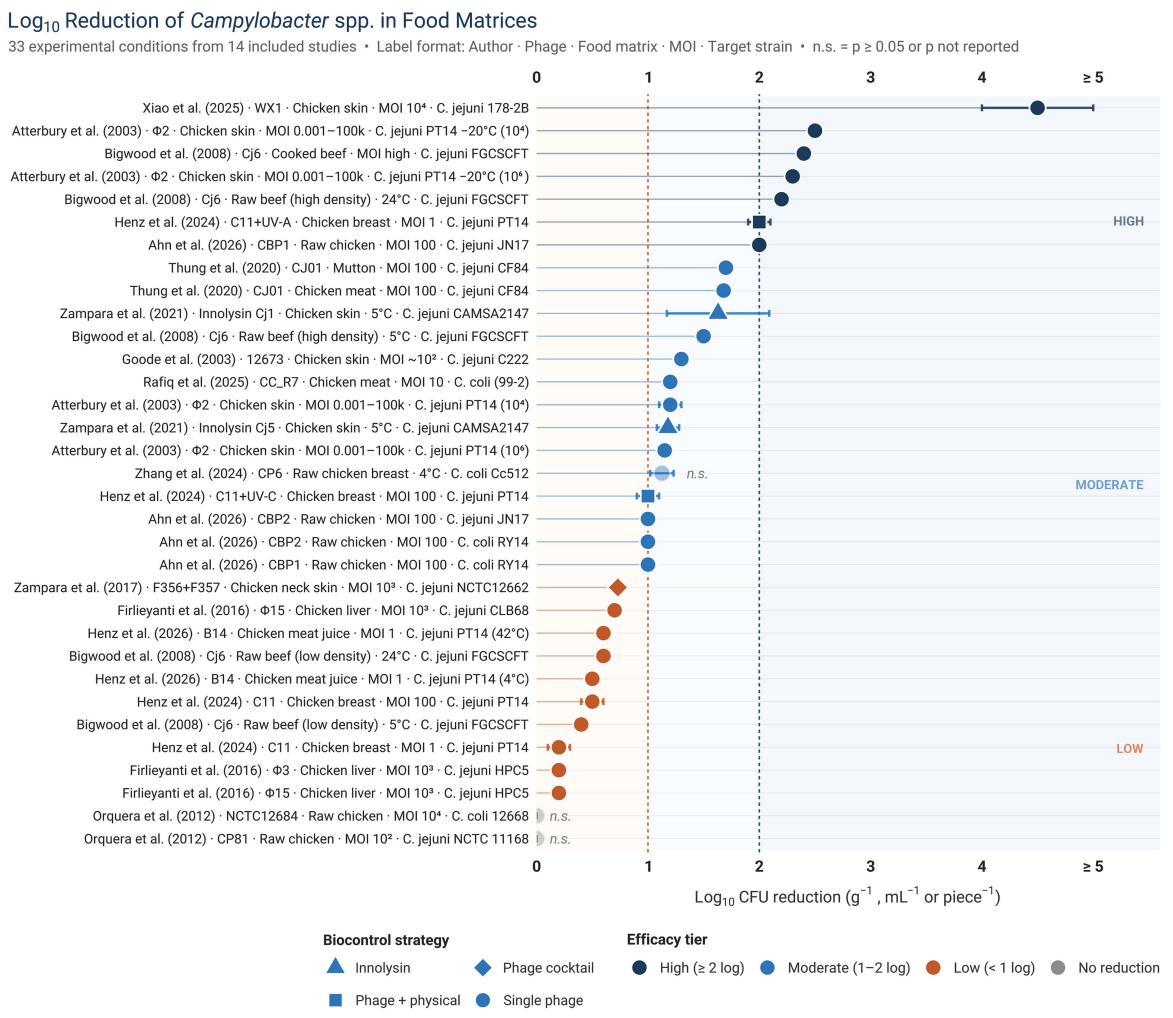
Log_10_ reduction of *Campylobacter* spp. in food matrices achieved by bacteriophage-based biocontrol strategies across included studies. Each data point represents one experimental condition reported across the 14 included studies (21 experimental conditions total). Horizontal bars indicate the reported range of reduction values; filled symbols denote the central or representative value. Symbol shape indicates the biocontrol strategy applied: circle = single phage; triangle (upward) = Innolysin; triangle (downward) = phage + UV-C; square = phage + UV-A; diamond = phage cocktail; asterisk = phage + physical method (freezing). Symbol color reflects the efficacy tier: navy = High (≥2 log_10_ CFU reduction); steel blue = Moderate (1-2 log_10_); orange-red = Low (<1 log_10_); grey = no reduction. Dashed vertical lines indicate the 1 log_10_ practical threshold (orange) and the 2 log_10_ regulatory benchmark referenced by EFSA (navy). Atterbury et al. (11), and Orquera et al. (21), each appear twice, representing distinct temperature conditions (4 °C and −20 °C; 4 °C and 37 °C, respectively); Ahn et al. (6), appears twice representing individual phages CBP1 and CBP2 tested against *C. jejuni* (CC-21) and *C. coli* (CC-828), respectively. n.s., reduction not statistically significant (*p* ≥ 0.05) or *p*-value not reported.

The magnitude of bacteriophage-mediated reductions of *Campylobacter* spp. in food matrices varied considerably across studies, ranging from 0.2 log_10_ to ~4.0–5.0 log_10_ CFU/mL, CFU/g or CFU/piece, depending on phage type, food matrix, bacterial inoculum, MOI, and storage conditions (Figure 3). This wide range of outcomes indicates that phage efficacy is highly dependent on the experimental context and should not be generalized across food matrices or storage conditions without caution. Studies conducted under refrigeration, in complex matrices, or under conditions that limit phage-host contact generally showed lower reductions, whereas higher reductions were observed mainly under optimized conditions that favored phage adsorption or were combined with additional physical interventions.

The highest reduction was achieved by Xiao et al. (12), who reported that phage WX1 reduced *C. jejuni* on chicken skin to below the detection limit, corresponding to an estimated reduction of ≥ 4–5 log_10_ CFU (*p* < 0.0001). Atterbury et al. (11), also reported substantial reductions under freezing conditions (−20 °C), with reductions of 2.3–2.5 log_10_ CFU on chicken skin when phage treatment was combined with freezing, compared to 1.1–1.3 log_10_ CFU under refrigeration (4 °C) alone (*p* ≤ 0.0001).

Intermediate reductions in the range of 1.0–2.4 log_10_ CFU were reported by Ahn et al. (6), (~2.0 log_10_ for *C. jejuni*, ~1.0 log_10_ for *C. coli*), Bigwood et al. (16), (up to 2.4 log_10_ on cooked beef, 1.5 log_10_ on raw beef), Goode et al. (20), (~1.3 log_10_ on chicken skin), Henz et al. (18), (2.0 log_10_ with phage + UV-A combined treatment), Orquera et al. (21), (1.0–2.0 log_10_ at 37 °C only), Rafiq et al. (22), (1.2 log_10_ CFU/g), Thung et al. (17), (1.68–1.70 log_10_ CFU/g), and Zhang et al. (5), (~1.0 log_10_ after 6 h). Zampara et al. (15), achieved reductions of 1.18–1.63 log_10_ using Innolysins on chicken skin.

The lowest reductions were reported by Firlieyanti et al. (14), where phages φ3 and φ15 yielded only 0.2–0.7 log_10_ CFU/g in chicken liver homogenates at 4 °C, and by Zampara et al. (19), whose phage cocktail (F356 + F357) achieved a maximum of 0.73 log_10_ CFU/g on chicken neck skin at 5 °C under modified atmosphere packaging. Notably, Henz et al. (2), reported that phage B14 efficacy in chicken meat juice was highly dependent on atmospheric and chemical conditions, with reductions ranging from 0.3 to 1.6 log_10_ CFU under varying oxygen concentrations and pH levels.

#### 3.4.1 Efficacy of phage biocontrol against MDR *Campylobacter* isolates

The application of bacteriophages against MDR *Campylobacter* isolates was investigated in studies where resistance profiles were either experimentally confirmed or cited from prior characterizations. Rafiq et al. (22), and Zhang et al. (5), conducted specific antimicrobial susceptibility testing within their study scope, identifying MDR prevalence rates of 85–95 and 65% among their isolated collections, respectively. In these experimental settings, phage CC_R7 reduced MDR contamination in chicken meat by 1.2 log_10_ CFU/g, while phage CP6 achieved reductions of ~1 log_10_ after 6 h in host cell counts on chicken breast meat. Conversely, other studies utilized host strains with MDR status established through characterizations in previous literature WX1 was applied against the cited MDR strain *C. jejuni* 178-2B, reducing bacterial counts on chicken skin to undetectable levels within 48 h. Similarly, phage CJ01 was evaluated against the characterized MDR isolate *C. jejuni* CF84, promoting reductions of 1.68 log_10_ CFU/g in chicken meat and 1.70 log_10_ CFU/g in mutton after 48 h.

### 3.5 Comparative properties of therapeutic phages

#### 3.5.1 Taxonomic classification

The 14 included studies characterized *Campylobacter* phages across different taxonomic and functional groups (Supplementary Table 1). According to the current ICTV taxonomy, the main classified *Campylobacter* phages reported in the included studies belong to the class *Caudoviricetes*, family *Connertonviridae*, and are primarily represented by the genera *Firehammervirus* and *Fletchervirus. Firehammervirus* corresponds broadly to Group II *Campylobacter* phages, whereas *Fletchervirus* corresponds broadly to Group III *Campylobacter* phages. Five studies reported phages assigned to *Firehammervirus*, one study reported phages assigned to *Fletchervirus*, and two studies included representatives of both genera. Three studies described Myovirus-like or myovirus morphotype phages without providing sufficient genomic information for updated genus-level assignment. Zampara et al. (15), applied engineered Innolysins, which are phage-derived proteins and therefore not classifiable as intact phages, while one study used an unclassified *Campylobacter* typing phage.

#### 3.5.2 Receptor specificity

Receptor specificity was characterized in five of the 14 included studies. Flagellar receptors were reported as the primary target between the studies (Supplementary Table 1). Additionally, capsular polysaccharide (CPS), specifically the MeOPN modification, was the target receptor for Group III phages, and CPS-dependent binding was confirmed for phage C11. Zhang et al. (5), reported tail fiber proteins, specifically the tail-fiber attachment catalyst CP6_069 and tail lysozyme CP6_164 as mediators of infection for phage CP6, though the specific surface receptor was not experimentally identified. Xiao et al. (12), associated WX1 infection with reduced flagellar development without confirming a specific receptor, and the Innolysin studies (15) targeted a novel prophage-derived receptor distinct from CPS or flagella, which remains unidentified.

### 3.6 Advanced biocontrol strategies and synergistic technologies

#### 3.6.1 Single vs. phage cocktails

Of the 14 included studies, 11 evaluated single phages and one evaluated a phage cocktail (Supplementary Table 1). Zampara et al. (19), evaluated both individual phages (F347–F388) and a two-phage cocktail (F356 + F357) on chicken neck skin at 5 °C. Individual phage applications yielded statistically non-significant reductions, whereas the cocktail produced a significant 0.73 log_10_ CFU/g reduction (*p* < 0.05), with a synergistic effect confirmed over each component phage individually (F356: *p* = 0.02; F357: *p* = 0.03).

#### 3.6.2 Combined treatments

Two studies evaluated combined phage-physical treatment strategies (Supplementary Table 1). Henz et al. (18), combined phage C11 with UV-A (286 s per 240 cm^2^ filet) and UV-C irradiation on chicken breast filets at 4 °C under microaerobic conditions. The additive synergistic effect between phage and UV-A yielded a 2.0 ± 0.1 log_10_ CFU/piece reduction, the highest among the combined and single-phage approaches at refrigeration temperature, compared to 0.5 ± 0.1 log_10_ for phage alone and 1.0 ± 0.1 log_10_ for phage + UV-C (*p* < 0.05). Atterbury et al. (11), demonstrated that freezing (−20 °C) combined with phage treatment produced synergistic reductions of 2.3–2.5 log_10_ CFU, significantly exceeding the reductions observed at 4 °C alone (1.1–1.3 log_10_).

#### 3.6.3 Biofilm eradication

Two studies reported on the anti-biofilm activity of the evaluated phages (Supplementary Table 1). Rafiq et al. (22), demonstrated that phage CC_R7 is genomically safe and capable of inhibiting and eradicating established *Campylobacter* biofilms under conditions mimicking slaughterhouse environments. Xiao et al. (12), reported significant biofilm reduction by phage WX1 on stainless-steel, polyethylene, and glass surfaces, relevant to food processing environments.

#### 3.6.4 Innolysins and endolysins

Zampara et al. (15), evaluated Innolysins, applied at a dose of 200 μg per chicken skin piece under modified atmosphere conditions at 5 °C for 45 min. Innolysin Cj1 achieved a reduction of 1.63 ± 0.46 log_10_ and Cj5 achieved 1.18 ± 0.10 log_10_ (both *p* < 0.05). These reductions exceeded those achieved by the replicating phage cocktail in the same laboratory (0.73 log_10_) (20).

## 4 Discussion

This systematic review synthesized evidence from 14 original peer-reviewed studies published between 2003 and 2026, collectively demonstrating that lytic bacteriophages constitute a biologically active and experimentally promising strategy for the biocontrol of *Campylobacter*, particularly in poultry products. Overall, 12 of the 14 studies reported statistically significant bacterial reductions in different experimental protocols, opening different types of bacteriophages, food matrices, temperature conditions, and geographical origins, reinforcing the potential of phage-based biocontrol as an emerging and complementary strategy for the strengthening of food safety (23).

The considerable variability in reported efficacy demonstrated that bacteriophage-mediated biocontrol of *Campylobacter* is highly context-dependent and cannot be evaluated through a single-condition framework. This heterogeneity, rather than representing inconsistency, reflects the complex interplay between phage biology, host physiology, and food matrix characteristics, each of which must be independently optimized for industrial translation. The high MOI and the permissive physicochemical environment for phage adsorption resulted in the greatest antibacterial efficacy by favoring phage–host interactions. Under these conditions, phage WXI reduced *C. jejuni* on chicken skin to below detectable levels (≥4-5 log_10_ CFU (12), while phage φ2 combined with freezing achieved reductions of 2.3–2.5 log_10_ CFU on chicken skin (11). In contrast, the lowest reductions ranging from 0.2 to 0.73 log_10_ were observed in matrices with unfavorable ecological conditions for phage activity, including matrices characterized by limited bacterial metabolic activity at refrigeration temperatures, such as chicken neck skin stored at 5 °C (19), or structurally complex tissues, such as chicken liver homogenates at 4 °C, where diffusion barriers and matrix composition may hinder effective phage-host contact (14).

From a food safety regulatory perspective, a minimum 2-3 log_10_ reduction is generally considered necessary to achieve meaningful risk reduction for *Campylobacter* in poultry (3), a threshold consistently achieved by only a minority of the included studies under realistic cold-storage conditions, indicating that although bacteriophage-based interventions are highly promising, their practical implementation in commercial food systems will likely require optimization strategies, including high-titer formulations, phage cocktails, or combined physical interventions.

### 4.1 Bacteriophage-mediated biocontrol of *Campylobacter* spp. accordingly food matrices

Chicken meat and skin were the most widely explored food matrices, being evaluated in 13 of the 14 studies included in this review. This high predominance of studies involving chicken meat is due to its frequent association as a major vehicle of human *Campylobacter* infection. *C. jejuni* and *C. coli* can reach concentrations of up to 8 log_10_ CFU/g in the intestinal tract of poultry, and carcass contamination commonly occurs during evisceration (24, 25). In parallel, bacteriophages targeting *Campylobacter* are consistently isolated from raw chicken meat and fecal samples, indicating their natural ecological association with MDR host strains within poultry production environments (5, 6, 12, 17, 22).

According to the most recent surveillance data, campylobacteriosis is the most frequently reported foodborne illness in EU, with over 246,000 human cases recorded annually, with *C. jejuni* and *C. coli* responsible for 87.5 and 11.9% of confirmed infections, respectively, confirming *C. jejuni* as the predominant causative agent of human campylobacteriosis (26). Although poultry is the primary transmission route, the prevalence of *Campylobacter* in other matrices is also significant and justifies its investigation; for example, in cattle, the frequency of the pathogen in muscle meat is generally very low, although Noormohamed and Fakhr (27) reported a high frequency of 78% in beef livers in the United States. Regional variations in prevalence are further documented in Australia, where Walker et al. (28), identified more moderate rates in offal samples, including 14% in beef, 31% in pork, and 38% in lamb, electing to sample organ meats because the pathogen is infrequently found in the muscle meat of beef, lamb, and pork. Strachan et al. (29), conducted a comprehensive investigation in the United Kingdom, evaluating multiple matrices simultaneously, which reported consistently high contamination levels across all tissues, finding a prevalence of 69% in cattle liver, 79% in pig liver, and 78% in sheep liver. Furthermore, in other poultry meats, such as duck and pigeon, isolation rates reach 29.6 and 38.2%, respectively (5).

Beef and mutton represent markedly underexplored matrices in the *Campylobacter* phage biocontrol literature. In this review, only one study evaluated each matrix. Thung et al. (17), evaluated that phage-based biocontrol in mutton was the first conducted on refrigerated retail lamb meat in Malaysia, achieving a 1.70 log_10_ CFU/g reduction with spray-applied CJ01. This reduction was comparable to that observed in chicken within the same study (1.68 log_10_), demonstrating that phage-based strategies can be successfully extended to non-poultry red meats with significant bacteriolytic effects. Bigwood et al. (16) demonstrated that phage Cj6 was effective on cooked beef (2.4 log_10_) but yielded more modest results on raw beef surfaces (1.5 log_10_), reflecting the influence of the matrix conditions on phage diffusion and contact. Overall, the limited number of studies beyond poultry matrices constitutes a significant knowledge gap that warrants prioritized investigation, considering the contribution of cattle and small ruminants to the global burden of campylobacteriosis.

### 4.2 Geographical and temporal distribution of studies

The geographical distribution of the analyzed studies reveals a strong predominance of Europe, holding 8 of the 14 articles (57.1%), followed by Asia with 5 contributions (35.7%), a pattern grounded in local regulatory and public health factors. In the EU, campylobacteriosis has been the most reported zoonosis since 2007 (26), and the rigor of European laws, which prohibit the chemical decontamination of carcasses with substances such as chlorine, drives the search for biological alternatives such as the phage biocontrol (30). Meanwhile, the recent growth in Asia, especially in China, is motivated by the critical emergence of MDR strains, with resistance rates to quinolones and tetracyclines exceeding 30% (31). Also, surveillance has revealed alarming rates, with reports indicating that 95% of *C. coli* and 85% of *C. jejuni* isolates are MDR, showing high resistance to tetracycline, nalidixic acid, and ciprofloxacin (5, 22).

The complete absence of contributions from South America and Africa is particularly noteworthy. Brazil is the world’s largest exporter of poultry meat, responsible for approximately 35% of global chicken exports in 2023, yet no studies on phage-based biocontrol of *Campylobacter* spp. in food matrices originating from this country were identified in the present review. This gap is unlikely to reflect a low disease burden; rather, it is consistent with the historically recognized underreporting of campylobacteriosis in Brazilian and broader South America surveillance systems, where clinical diagnosis is limited by the fastidious growth requirements of *Campylobacter* and the absence of mandatory national notification protocols (7). Future research efforts and international scientific cooperation should prioritize the generation of region-specific data from South America and Africa, where the substantial epidemiological burden is further linked to systemic underreporting, often compounded by precarious sanitation infrastructure and limited access to treated water, factors that further hinder the systematic documentation and control of the pathogen.

The temporal concentration of eight studies after the year 2020 is related to fundamental biotechnological and regulatory milestones. Advances in genomic sequencing tools and bioinformatics have allowed for the verification of the genetic safety of phages, proving the absence of genes associated with lysogeny, virulence factors or toxins and antibiotic resistance, much more accurately than in previous decades (32). Furthermore, a turning point occurred in 2025, when the European Commission authorized, for the first time, a phage preparation against *Campylobacter* as a feed additive for poultry (33), stimulating experimental research to validate this new commercial feasibility.

### 4.3 Taxonomic classification and genomic safety of therapeutic phages

The taxonomic classification of *Campylobacter* bacteriophages is not merely a matter of nomenclatural convention; it serves as a functional framework for predicting host range, receptor specificity, thermal behavior and genetic safety, parameters that collectively determine the suitability of a phage candidate for food-grade biocontrol applications. *Campylobacter* phages are primarily classified into three genomic groups: Group I, Group II and Group III, with most therapeutically relevant isolates belonging to the family *Myoviridae*, characterized by icosahedral capsids and contractile tails (6, 17).

Under the current ICTV taxonomy, classified *Campylobacter* phages previously described as Group II and Group III *myoviruses* are now placed within the class *Caudoviricetes* and family *Connertonviridae*, mainly in the genera *Firehammervirus* and *Fletchervirus*, respectively (6, 12). Although the historical Group II/Group III terminology remains useful for discussing functional differences in host range, receptor specificity, and thermal behavior, the updated ICTV nomenclature was adopted throughout the revised manuscript.

Group II phages (*Firehammervirus*, formerly *CP220-like viruses*), are morphologically distinguished by long contractile tails measuring approximately 110–142 nm in length (6, 22). At the genomic level, they are uniquely characterized by the presence of up to 12 proteins containing S-adenosylmethionine (SAM) domains, which are completely absent in Group III members (22). Furthermore, Group II genomes frequently harbor numerous transposases and homing endonucleases, for instance, phage CC_R7 contains 9 transposases, suggesting a high potential for genomic rearrangements and instability (5, 22). From a biocontrol perspective, the principal advantage of Group II phages lies in their dual-species activity: their flagellotropic infection mechanism, which requires contact with motile bacterial flagella, enables them to infect both *C. jejuni* and *C. coli* across phylogenetically diverse lineages (5, 6, 22). This broad-spectrum coverage is particularly relevant against the globally dominant clonal complexes CC-21 (*C. jejuni*) and CC-828 (*C. coli*), both of which are frequently associated with antibiotic resistance and human infection (6).

Group III phages (*Fletchervirus*, formerly *Cp8unalikeviruses*) present a contrasting biological profile. Morphologically characterized by significantly shorter contractile tails measuring approximately 98–110 nm in length, these phages exhibit a markedly narrower host range, infecting almost exclusively *C. jejuni* and remaining largely ineffective against *C. coli* (6, 17, 22). This restriction is mechanistically explained by their primary dependency on capsular polysaccharides (CPS) as receptor targets, with specificity for O-methyl phosphoramidate (MeOPN) modifications on the bacterial capsule (19). Despite this limitation, Group III phages possess a critical operational advantage in post-harvest settings: CPS-dependent binding is both more efficient and irreversible at standard refrigeration temperatures, in contrast to the reversible, motility-dependent adsorption of flagellotropic Group II phages under the same conditions (19). Group I phages, which possess the largest genomes of the three groups, remain rarely isolated and are currently insufficiently characterized to assess their potential for food safety applications (6).

From a practical biocontrol standpoint, the taxonomic dichotomy between Group II and Group III phages demonstrates a fundamental relationship with direct implications for product development. Group II phages offer broad-spectrum coverage against *C. jejuni* and *C. coli* and perform optimally under the high temperatures associated with active bacterial replication, while their flagelotropic dependence makes them substantially less effective at refrigeration temperatures, precisely the prevailing conditions during post-slaughter poultry processing and cold chain storage in retail. On the other hand, Group III phages exhibit superior irreversible binding efficacy above 4-5 °C, but are limited by a restricted host range, which restricts their commercial viability as isolated biocontrol agents. This biological complementarity, broad spectrum versus cold chain activity, provides the strongest mechanistic rationale for the development of multi-group cocktail formulations, in which phages from Groups II and III act synergistically to maximize efficacy across the thermal and taxonomic diversity inherent in commercial food matrices.

The genomic safety of phage candidates constitutes an equally indispensable criterion for regulatory approval in food applications. Comprehensive genomic analyses of therapeutically relevant phages, including CBP1, CBP2, CC_R7, WX1, and CP6, have confirmed the absence of genes encoding lysogeny functions, virulence factors, toxins, and antimicrobial resistance determinants (5, 6, 12, 22). The exclusive use of strictly lytic phages is a non-negotiable regulatory requirement for food-grade applications, as it eliminates the risk of horizontal gene transfer of pathogenic elements and prevents the integration of viral DNA into the host chromosome, a mechanism through which temperate phages could, in principle, contribute to the dissemination of virulence or resistance traits (6). Beyond host safety, the narrow host specificity of lytic phages confers an additional ecological advantage: their activity is inherently self-limiting in the absence of a susceptible host, minimizing unintended perturbation of the consumer’s gut microbiome and the broader food ecosystem (5, 12). Together, these properties position well-characterized, strictly lytic *Campylobacter* phages as scientifically sound tractable candidates for integration into evidence-based food safety frameworks.

### 4.4 Influence of food matrix and physicochemical conditions on phage efficacy

The predominance of chicken meat as a matrix in 13 of the 14 studies is justified by the fact that poultry are the primary reservoir of the pathogen, being responsible for 50 to 80% of human campylobacteriosis cases (34). Poultry carry extreme bacterial loads in the cecum, reaching 6–8 log_10_ CFU/g in young chicks (35), which facilitates dissemination to the meat during critical stages such as evisceration and defeathering (6). In high-contamination matrices like chicken, reductions in the range of 0.2 to 0.7 log_10_ CFU/g are insufficient and chicken-derived group III phages often exhibit narrow host ranges (14). Although cattle, sheep, and pigs are known reservoirs (29), these matrices remain less explored due to the specific processing characteristics associated with their food production. In pigs, for example, carcass processing involves chilling stages, such as blast chilling, that utilize forced ventilation to cause surface desiccation, a condition to which *Campylobacter* is highly susceptible (36). This drying process, combined with oxygen exposure, reduces the bacterial load significantly, often to undetectable levels, which may represent a more drastic reduction compared to the survival rates typically observed in the chicken chilling stages.

Regarding phage delivery, the entire surface area of the meat and carcasses was sprayed using small dispensers across most studies, as this is recognized as an efficacious and standardized delivery method for phage-based interventions (17, 20). Experimental protocols were generally conducted under refrigeration conditions, specifically at 4 °C or 5 °C, to simulate realistic retail and domestic storage (5, 11, 16, 17, 19).

Although pH was not systematically varied across most included studies, which focused on realistic retail simulation, Henz et al. (2) provided the most mechanistic characterization of physicochemical determinants, demonstrating that phage efficacy was most efficient at 7.4, while at pH 6, common on meat surface, activity was restricted. Beyond temperature and pH, retail conditions such as high-oxygen atmospheres (70–80% O_2_), acidic pH, low temperatures, and meat juice particles can further reduce efficacy by physically blocking phage attachment to bacterial surfaces (2).

The variability in efficacy outcomes across food matrices and storage conditions represents a major limitation for generalizing the findings of this review. Phage-mediated reductions were not uniform among studies and ranged from modest decreases under refrigeration or structurally complex matrices to higher reductions under optimized experimental conditions. This variability reflects the influence of matrix composition, surface topology, organic matter, bacterial physiological state, MOI, exposure time, pH, oxygen concentration, and temperature on phage adsorption and host-cell contact. Refrigeration temperatures may restrict *Campylobacter* metabolic activity and limit phage replication, while irregular food surfaces such as chicken skin follicles, folds, and lipid-rich areas may physically protect bacterial cells from phage access. Therefore, efficacy results should be interpreted in a matrix-specific and condition-specific manner rather than as broadly generalizable across all food systems.

The discrepancy in log reduction results, which range from 0.2 to approximately 4.0–5.0, is primarily attributed to variations in temperature, MOI, and the nature of the food matrix. The most significant biological barrier is that *Campylobacter* does not replicate at refrigeration temperatures, which restricts phages to a single infection cycle or non-proliferative lysis, limiting post-harvest efficacy (6, 19). Consequently, studies conducted under refrigeration typically yield lower reductions (0.5 to 1.5 log_10_), whereas the highest results are generally observed only under optimized growth conditions (37 °C–42 °C) where phages can actively propagate and reduce the counts to undetectable levels. Flagellotropic phages such as B14 are particularly affected by this constraint, exhibiting reversible binding and impaired irreversible attachment in non-growth environments (2, 19). Notably, B14 is uniquely characterized by the lack of hypermutable polyG-tracts, a genomic feature that avoids generation-dependent host evasion often observed in capsular polysaccharide-dependent phages (2).

The complexity of the matrix plays a critical role; while phages diffuse more easily in liquid models or smooth meat surfaces, the anatomy of chicken skin, characterized by follicles, folds, and oils, acts as a physical shield that protects bacteria from phage contact. Despite physical barriers, *Campylobacter* survivors on treated chicken skin remained sensitive and did not differ significantly to the original strain (11). High MOIs, generally ranging from 100 to 1,000, are often required to overcome physical barriers and achieve significant reductions, potentially resulting in an approximately 95% reduction in recovered bacterial counts (20).

### 4.5 Host specificity, phage selection, and bacterial resistance mechanisms

A primary biological limitation is the narrow host specificity of many isolated phages, such as Cj6 (16) and CLP6, CLP47, and CLP63 (14), which often fail to infect the diverse *Campylobacter* genotypes coexisting in poultry production (6). The lytic rate of phage WX1, for example, reached only 47.3% across the 76 strains tested (12), despite demonstrating remarkable efficacy by reducing *C. jejuni* counts on chicken skin to undetectable levels within 48 h under optimized conditions; additionally, WX1 is highly dependent on the host’s translational machinery due to a lack of encoded tRNAs, which may further restrict its broader applicability. Although bacterial defense through restriction-modification systems is possible, it has been demonstrated that high doses of phages can overcome this resistance via “lysis from without” (20). Dual-species activity against predominant lineages like CC-21 and CC-828 remains rare, necessitating the continuous development of complex, broad-spectrum cocktails (6). Strategically combining group II and III phages is essential to prevent resistance, particularly against invasive strains like CLB104, that translocate to extra-intestinal organs and resist natural decline during storage (5, 14). Furthermore, certain group II phages harbor numerous genes for transposases and homing endonucleases, indicating a potential for genetic instability (5).

An important advantage of phage-based biocontrol is that the lytic efficacy of phages is generally independent of the host’s antibiotic resistance profile, making them particularly relevant against MDR strains (12). The novel phage CP6 demonstrated an exceptionally broad host range, infecting 97.4% of *Campylobacter* isolates tested, including 33 out of 34 MDR strains (5). Similarly, broad-host-range phages CBP1 and CBP2 proved effective against globally significant lineages such as CC-21 (*C. jejuni*) and CC-828 (*C. coli*), which are frequently associated with both antibiotic resistance and human infection, with CBP1 and CBP2 inhibiting 70.8 and 75% of *C. coli* isolates, respectively (6).

The observation that some isolated phages demonstrated higher efficacy than cocktails is likely due to receptor specificity and differing study conditions. *In situ* studies on chicken skin revealed that while single CPS-dependent phages F356 and F357 achieved reductions of only 0.40 to 0.43 log_10_ units individually, their combination in a cocktail increased the reduction to 0.73 log_10_ units. Group III CPS-dependent phages bind more efficiently and irreversibly at low temperatures compared to flagellotropic phages, though their killing efficiency on solid matrices remains modest (19). Although antibacterial cocktails broaden the lytic spectrum, mitigate bacterial heterogeneity, and prevent resistance through phase variation, their use in research is sometimes limited to avoid complexity when investigating specific environmental factors, and because maintaining the stability of multiple phages in a single commercial formulation remains a practical and regulatory challenge.

The emergence of phage-resistant mutants and host regrowth, observed as early as 21 h post-infection, represents a significant disadvantage. *Campylobacter* populations adapt through phase variation of surface receptors such as flagella or capsular polysaccharides, allowing them to evade phage infection. To counteract host restriction mechanisms, phage CC_R7 possesses a 7-carboxy-7-deazaguanine synthase enzyme that modifies phage DNA, shielding it from being targeted by bacterial restriction enzymes and thus enhancing its persistence within the host (22). Furthermore, the use of strictly lytic phages that target conserved receptors across phylogenetically diverse lineages reduces the probability of resistance emergence without initiating horizontal gene transfer (5, 6).

### 4.6 Biofilm inhibition and phage persistence on food contact surfaces

Isolated phages like WX1 and CC_R7 have shown a remarkable ability to decrease or inhibit biofilm formation on industrial surfaces such as stainless steel, polyethylene, and glass. This efficiency is due to their ability to penetrate the extracellular matrix and interrupt cell communication; WX1 maintained biomass levels below 0.2, compared to 1.0 for the control, by limiting flagellar development, which is essential for maintaining biofilm integrity. Furthermore, CC_R7, which is genomically safe, proved effective even under slaughterhouse conditions (10 °C and 42 °C), achieving superior performance at a MOI of 10^2^ and demonstrating the capacity to inhibit or even eradicate established *Campylobacter* biofilms (22). Notably, phages can persist on skin for 48 h, preventing cross-contamination, and survive up to 10 days independently of host growth (11, 20), representing a practical advantage for food safety applications.

### 4.7 Future perspectives

Innolysins are considered promising because these engineered enzymes act independently of host metabolism, targeting conserved receptors not associated with capsules or flagella, thus hindering resistance through phase variation. It was recently discovered that the H-fiber, originating from a CJIE1-like prophage in the *C. jejuni* CAMSA2147 strain, acts as a functional receptor-binding protein when co-expressed with a specific DUF4376-domain chaperone. The engineered Innolysin Cj1 achieved a 1.63 ± 0.46 log_10_ reduction of *C. jejuni* on refrigerated chicken skin at 5 °C, while Innolysin Cj5, which incorporates a *Salmonella* phage Shivani peptidase, achieved a 1.18 log_10_ reduction under similar *in situ* conditions (15). However, killing efficiency remains moderate, specific receptors remain unidentified, and the gram-negative outer membrane acts as a substantial physical barrier restricting the enzyme’s full access to the peptidoglycan layer. While resistance to engineered proteins like Innolysins is possible, it is typically limited to receptor mutations that may carry a significant biological disadvantage, such as reduced bacterial virulence. Simultaneous application targeting diverse receptors could further minimize bacterial adaptation risks (15).

Beyond Innolysins, the broader class of phage-derived lytic proteins represents a rapidly expanding frontier for food biocontrol. Virion-associated lysins (VALs) are gaining increasing attention as complementary enzymatic tools (37). However, these enzymes are typically embedded within phage tail structural components, requiring more intricate genetic and bioinformatic analysis for their discovery and characterization compared to endolysins (37). The identification and engineering of such proteins targeting *Campylobacter*-specific surface structures could substantially expand the enzymatic arsenal available for post-harvest biocontrol.

An additional avenue of significant promise involves the use of metagenomic approaches to mine novel phage-derived enzymes from uncultured environmental viruses, leveraging high-throughput sequencing data to identify candidates without the need for phage cultivation (37). This strategy could overcome the technical constraint of phage isolation from culturable hosts, particularly relevant for *Campylobacter*, given the fastidious nature of the bacterium under *in vitro* conditions.

The engineering of bacteriophages through CRISPR-Cas systems represents a transformative perspective for overcoming the current limitations of phage therapy. By equipping phages with the Type I-E CRISPR-Cas3 machinery, it is possible to move beyond simple lysis toward a sequence-specific DNA degradation that ensures a more robust and lethal attack on the target pathogen (38). Designing phages that can recognize independent surface components simultaneously directly addresses the narrow host range and the rapid emergence of resistance that often restrict the use of wild-type phages (38). Furthermore, the success of engineered platforms like SNIPR001 in achieving precise pathogen reduction without disturbing the surrounding microbial community provides a clear blueprint for food safety applications (38). This technology offers a promising path for developing broad-spectrum agents capable of simultaneously targeting the complex lineages of both *C. jejuni* and *C. coli*, effectively bridging the gap between clinical innovation and industrial biocontrol.

The encapsulation of bacteriophages within nano and microcarrier systems has emerged as a promising strategy to overcome the physicochemical limitations imposed by food matrices, such as acidic pH, high oxygen atmospheres, and matrix-mediated physical barriers. Biodegradable and biocompatible materials, including chitosan nanoparticles, alginate microcapsules, and liposomes, have demonstrated the capacity to protect phage virions from environmental degradation, improve delivery to bacterial targets, and enable sustained or controlled phage release throughout the product shelf life (37, 39). Although encapsulation studies specifically targeting *Campylobacter* in food matrices remain scarce, the results obtained for other foodborne pathogens, such as *Salmonella* and *Staphylococcus aureus*, suggest that this approach could significantly enhance the post-harvest efficacy of phage-based interventions under realistic cold-storage conditions (39).

The combination of treatments often produces additive or synergistic effects by creating multiple simultaneous obstacles for the bacteria. Physical treatments like UV-A laser or UV-C light cause direct DNA damage or mechanical stress to the bacterial membrane, making cells more vulnerable to subsequent phage attachment or lysis (18). While UV-A laser irradiation alone inactivates 1.8 log_10_, its association with the C11 phage increased the reduction to 2.0 log_10_. Conversely, UV-C lamps achieved a lower inactivation of 0.5 log_10_ units alone, which increased to 1.0 log_10_ units when combined with phage treatment, with this combined effect being primarily additive, allowing phages to act even when light penetration is limited by the meat matrix (18). Additionally, the combination of phage treatment and freezing has shown significant promise; higher concentrations of phages on frozen chicken skin resulted in reductions of up to 2.5 log_10_ units, with chicken skin follicles and oils appearing to offer partial protection to phage particles against ice crystal formation during the freezing process (11). However, industrial scalability remains a challenge, as UV-A laser protocols require approximately 286 s per piece, although they demonstrate superior efficacy over UV-C (18).

### 4.8 Methodological limitations and current commercial landscape

A limitation of this review is the relatively small number of eligible studies. Although the systematic search retrieved 432 records, only 14 studies met the inclusion criteria for evaluating phage-based post-harvest biocontrol of *Campylobacter* spp. in food matrices. Therefore, while the available evidence supports the experimental potential of lytic bacteriophages, broad conclusions regarding industrial applicability should be interpreted with caution. The limited number of studies, together with the predominance of poultry matrices, the scarcity of non-poultry food models, the heterogeneity of phage application protocols, and the lack of large-scale validation under commercial processing conditions, restricts the generalizability of the findings. Future studies should prioritize standardized experimental designs and industrial-scale trials under realistic cold-chain and food-processing conditions.

Methodologically, several limitations affect the generalizability of findings across the reviewed studies. Suppressed resident microbiota in controlled trials may mask natural interferences, while unrealistic experimental conditions, such as 24 h at 24 °C, and meat surface drying can restrict phage diffusion (6, 16). Liquid models at 42 °C do not reflect retail refrigeration, and some group II trials lacked dose-dependent activity. Chemical factors also interfere, with Mg^2+^ inhibiting phage activity, an effect countered by Ca^2+^.

A clear distinction should be made between laboratory efficacy and real-world applicability. Most studies included in this review were conducted under controlled experimental conditions, using defined bacterial inoculum, selected phage concentrations, simplified food matrices, and standardized storage settings. These designs are essential for determining proof-of-concept efficacy, but they do not fully reproduce the complexity of commercial slaughterhouse and food-processing environments. In industrial settings, phage performance may be affected by heterogeneous contamination levels, organic matter, carcass surface irregularities, resident microbiota, processing speed, spray coverage, temperature fluctuations, packaging atmosphere, sanitation procedures, and cold-chain duration. Therefore, the log reductions reported under laboratory conditions should be interpreted as indicators of experimental potential rather than direct evidence of industrial effectiveness.

Beyond study-specific constraints, a structural impediment lies in the considerable heterogeneity of experimental protocols across the phage biocontrol literature, including divergent phage titration methods, inconsistent reporting of MOI values, and the absence of standardized efficacy benchmarks, which collectively impede cross-study comparisons and the synthesis of reproducible, evidence-based guidelines for industrial application (23). To overcome these gaps, global implementation will require harmonized data standards and standardized dossier formats, alongside standardized genomic characterization, verified through whole-genome sequencing (WGS), that supports interoperable dossier cores and the synthesis of a minimum evidence package ready for industrial application (23).

An additional barrier is the limited understanding of phage interactions with the human host. Zamora et al. (40) has identified that bacteriophages can penetrate airway epithelial cells and elicit diverse immune responses, highlighting the need for further investigation of phage-human interactions to establish comprehensive safety guidelines for food-grade applications. Furthermore, the potential sensory effects of phage-derived proteins on food quality, including possible enzymatic interactions with food components such as proteins, fats, and carbohydrates, which could lead to unintended alterations in taste, texture, color, and aroma, remain insufficiently characterized (41), representing a critical knowledge gap for consumer acceptance and industrial feasibility.

From an implementation perspective, additional validation is required before phage-based interventions can be broadly recommended for commercial food-processing systems. Future studies should evaluate phage formulations under pilot-scale and industrial-scale conditions, including realistic contamination scenarios, high-throughput application methods, formulation stability, compatibility with existing processing steps, sensory effects on food products, regulatory requirements, and cost-effectiveness. Such studies are necessary to determine whether the promising reductions observed in laboratory models can be consistently reproduced under commercial conditions.

Currently, commercial availability remains limited to GRN 966 (*Campyshield*™) in the United States and a single authorized feed additive in the EU. The regulatory landscape varies notably between countries: while phage products have not yet received widespread approval for direct food application throughout the EU, they are already being used in the food sector in countries such as the United States, Canada, Israel and Netherlands. In the United States, bacteriophages are frequently classified by the FDA as Generally Recognized as Safe (GRAS), with *Campyshield*™ representing the only approved product specifically targeting *Campylobacter* for use in both meats and food processing equipment. More broadly, regulatory oversight of phage-based food products is typically implemented through existing national frameworks, such as feed additives, veterinary products, or biopesticides, rather than through harmonized, phage-specific food legislation, resulting in authorizations that are country-specific and heterogeneous. The same phage preparation may be categorized as a processing aid, a food additive, or a biopesticide depending on the target market, creating significant regulatory fragmentation that hinders global commercialization (23). The lack of public awareness remains a key barrier to the widespread implementation of bacteriophages across the One Health perspective. These gaps underscore the persistent disparity between experimental progress and practical implementation at industrial scale.

### 4.9 Knowledge gaps and future research priorities

Despite the promising experimental evidence supporting the use of lytic bacteriophages for the post-harvest biocontrol of *Campylobacter* spp., several knowledge gaps remain and currently limit broader generalization and industrial implementation. First, the available evidence is based on a limited number of eligible studies, most of which were conducted under controlled laboratory conditions rather than under pilot-scale or commercial food-processing environments. Therefore, additional validation is required to determine whether the reductions observed in experimental models can be consistently reproduced under realistic slaughterhouse, processing, packaging, and cold-chain conditions.

Second, the current literature is strongly concentrated on poultry matrices, particularly chicken skin and meat, while non-poultry food matrices such as beef, mutton, offal, and other animal-derived products remain underexplored. This limits the extrapolation of efficacy outcomes across different food systems, especially because phage performance is strongly influenced by matrix composition, surface structure, organic matter, pH, oxygen concentration, temperature, and storage time. Future studies should therefore include matrix-specific validation using experimentally standardized but commercially realistic models.

Third, substantial methodological heterogeneity persists across studies, including differences in bacterial inoculum, phage dose, MOI reporting, application method, exposure time, temperature, packaging atmosphere, and outcome measurement. This variability hampers direct comparison among studies and limits the establishment of evidence-based efficacy benchmarks. Future research should adopt harmonized reporting standards, including detailed descriptions of bacterial strains, phage identity, genomic safety, application protocols, replication structure, statistical analysis, and log-reduction endpoints.

Fourth, further investigation is needed to optimize phage formulations for practical application. Multi-phage cocktails combining complementary host ranges and receptor specificities may improve coverage against diverse *Campylobacter* populations and reduce the risk of resistance emergence. However, cocktail stability, formulation compatibility, delivery efficiency, and performance during refrigerated storage require additional study. Encapsulation and other protective delivery systems also represent promising strategies to improve phage stability and persistence in complex food matrices.

Fifth, the sensory, technological, and regulatory aspects of phage-based interventions remain insufficiently characterized. Future studies should evaluate whether phage preparations or phage-derived proteins affect the sensory properties, shelf life, or technological quality of food products. In parallel, more harmonized regulatory frameworks are needed to support consistent safety assessment, approval, and implementation of phage-based products across different jurisdictions.

Finally, long-term monitoring of bacterial resistance to phages should be incorporated into future experimental and applied studies. Although strictly lytic phages are promising tools for targeting *Campylobacter* independently of antimicrobial resistance profiles, the emergence of phage-resistant variants through receptor modification, phase variation, or other adaptive mechanisms remains a relevant concern. Future research should therefore integrate resistance monitoring, genomic surveillance, and assessment of phage-host coevolution into the development of phage-based biocontrol strategies.

## 5 Conclusion

This systematic review indicates that lytic bacteriophages represent a promising experimental strategy and an emerging post-harvest biocontrol approach for reducing *Campylobacter* spp. in food matrices, with statistically significant reductions reported in most of the included studies. However, the magnitude of efficacy varied considerably across experimental conditions, depending on factors such as food matrix, storage temperature, bacterial inoculum, MOI, phage formulation, exposure time, and the use of combined interventions.

Under refrigeration conditions, which are more representative of post-harvest storage and cold-chain scenarios, most studies reported modest reductions, generally around 0.5–1.5 log_10_ CFU. Reductions within the 2-3 log_10_ range considered more relevant for meaningful food safety risk reduction were achieved only in a limited number of studies, mainly under optimized experimental conditions or when phage application was combined with physical interventions such as freezing or UV treatment.

Importantly, the available evidence is based on a limited number of eligible studies, with only 14 studies meeting the inclusion criteria. Therefore, broad conclusions regarding industrial applicability should be interpreted with caution. The predominance of poultry-based matrices, the limited number of studies involving non-poultry foods, the heterogeneity of phage application protocols, and the scarcity of large-scale trials under commercial processing conditions restrict the generalizability of the findings.

A relevant advantage of phage-based biocontrol is that lytic activity may occur independently of the antibiotic resistance profile of the bacterial host, supporting its potential relevance against multidrug-resistant *Campylobacter* strains. Emerging strategies, including Innolysins, phage cocktails, and combined physical-biological treatments, appear promising for overcoming some of the thermal and matrix-related limitations observed with single-phage applications. Nevertheless, these approaches still require further validation under standardized and commercially relevant conditions.

Overall, the findings support the potential of bacteriophages as complementary tools for improving post-harvest *Campylobacter* control in food systems. Future studies should prioritize standardized efficacy metrics, harmonized reporting of MOI and experimental conditions, diverse food matrices, realistic cold-chain scenarios, long-term resistance monitoring, sensory and technological assessments, and industrial-scale validation before broader implementation in commercial food-processing environments can be fully supported.

## Data Availability

The original contributions presented in the study are included in the article/Supplementary material, further inquiries can be directed to the corresponding author.

## Supplementary material

SUPPLEMENTARY TABLE 1 Comparative characterization of bacteriophages and phage-derived biocontrol strategies evaluated against *Campylobacter* spp. in post-harvest food matrices across the studies included in this systematic review. The table summarizes phage designation, taxonomic classification, host range, target receptors, food matrices, multiplicity of infection (MOI), storage conditions, antibacterial efficacy (log_10_ reduction), combined treatments, anti-biofilm activity, genomic safety features, and principal experimental outcomes reported in each study.

## References

[1] Havelaar AH Kirk MD Torgerson PR Gibb HJ Hald T Lake RJ . World Health Organization global estimates and regional comparisons of the burden of foodborne disease in 2010. PLoS Med. (2015) 12:e1001923. doi: 10.1371/journal.pmed.1001923 26633896 PMC4668832

[2] Henz S Nitzsche R Stukenborg L Aganovic K Hertel C. Food-related factors influencing phage efficacy in reducing *Campylobacter jejuni* counts. Food Microbiol. (2026) 134:104930. doi: 10.1016/j.fm.2025.104930, 41136147

[3] EFSA Panel on Biological Hazards (BIOHAZ). Scientific opinion on Campylobacter in broiler meat production: control options and performance objectives and/or targets at different stages of the food chain. EFSA J. (2011) 9:2105. doi: 10.2903/j.efsa.2011.2105 42078736 PMC13129689

[4] Hoffman S Ahn J-W. Updating Economic Burden of Foodborne Diseases Estimates for Inflation and Income Growth. Washington, DC: United States Department of Agriculture (2021).

[5] Zhang X Tang M Zhou Q Lu J Zhang H Tang X . A broad host phage, CP6, for combating multidrug-resistant Campylobacter prevalent in poultry meat. Poult Sci. (2024) 103:103548. doi: 10.1016/j.psj.2024.103548, 38442560 PMC10964072

[6] Ahn E Kim J Kim J Hur JI Ryu S Jeon B. Broad-host-range bacteriophages CBP1 and CBP2 with dual-species activity against *Campylobacter jejuni* and *Campylobacter coli*. Int J Food Microbiol. (2026) 444:111469. doi: 10.1016/j.ijfoodmicro.2025.111469, 41037991

[7] Epps S Harvey R Hume M Phillips T Anderson R Nisbet D. Foodborne Campylobacter: infections, metabolism, pathogenesis and reservoirs. Int J Environ Res Public Health. (2013) 10:6292–304. doi: 10.3390/ijerph10126292, 24287853 PMC3881114

[8] European Food Safety Authority and European Centre for Disease Prevention and Control (EFSA and ECDC). The European Union summary report on trends and sources of zoonoses, zoonotic agents and food-borne outbreaks in 2017. EFSA J. (2018) 16:e05500. doi: 10.2903/j.efsa.2018.5500 32625785 PMC7009540

[9] Taha-Abdelaziz K Singh M Sharif S Sharma S Kulkarni RR Alizadeh M . Intervention strategies to control Campylobacter at different stages of the food chain. Microorganisms. (2023) 11:113. doi: 10.3390/microorganisms11010113, 36677405 PMC9866650

[10] Atterbury RJ Connerton PL Dodd CER Rees CED Connerton IF. Application of host-specific bacteriophages to the surface of chicken skin leads to a reduction in recovery of *Campylobacter jejuni*. Appl Environ Microbiol. (2003) 69:6302–6. doi: 10.1128/AEM.69.10.6302-6306.2003, 14532096 PMC201188

[11] Atterbury RJ Connerton PL Dodd CER Rees CED Connerton IF. Isolation and characterization of Campylobacter bacteriophages from retail poultry. Appl Environ Microbiol. (2003) 69:4511–8. doi: 10.1128/AEM.69.8.4511-4518.2003, 12902236 PMC169066

[12] Xiao K Pan Q Wu Y Ding Y Wu Q Zhang J . Application of a novel phage vB_CjeM_WX1 to control *Campylobacter jejuni* in foods. Int J Food Microbiol. (2025) 427:110975. doi: 10.1016/j.ijfoodmicro.2024.110975, 39550792

[13] Page MJ McKenzie JE Bossuyt PM Boutron I Hoffmann TC Mulrow CD . The PRISMA 2020 statement: an updated guideline for reporting systematic reviews. BMJ. (2021) 372:n71. doi: 10.1136/bmj.n71, 33782057 PMC8005924

[14] Firlieyanti AS Connerton PL Connerton IF. Campylobacters and their bacteriophages from chicken liver: the prospect for phage biocontrol. Int J Food Microbiol. (2016) 237:121–7. doi: 10.1016/j.ijfoodmicro.2016.08.026, 27565524 PMC5064024

[15] Zampara A Sørensen MCH Gencay YE Grimon D Kristiansen SH Jørgensen LS . Developing innolysins against *Campylobacter jejuni* using a novel prophage receptor-binding protein. Front Microbiol. (2021) 12:619028. doi: 10.3389/fmicb.2021.619028, 33597938 PMC7882524

[16] Bigwood T Hudson JA Billington C Carey-Smith GV Heinemann JA. Phage inactivation of foodborne pathogens on cooked and raw meat. Food Microbiol. (2008) 25:400–6. doi: 10.1016/j.fm.2007.11.003, 18206783

[17] Thung TY Lee E Mahyudin NA Wan Mohamed Radzi CWJ Mazlan N Tan CW . Partial characterization and in vitro evaluation of a lytic bacteriophage for biocontrol of *Campylobacter jejuni* in mutton and chicken meat. J Food Saf. (2020) 40:e12770. doi: 10.1111/jfs.12770

[18] Henz S Nguyen MTT Nitzsche R Polley J Harmeling H Aganovic K . UV-A-laser or UV-C irradiation combined with bacteriophage treatment to combat *Campylobacter jejuni* on chicken breast fillets. Innov Food Sci Emerg Technol. (2024) 95:103740. doi: 10.1016/j.ifset.2024.103740

[19] Zampara A Sørensen MCH Elsser-Gravesen A Brøndsted L. Significance of phage-host interactions for biocontrol of *Campylobacter jejuni* in food. Food Control. (2017) 73:1169–75. doi: 10.1016/j.foodcont.2016.10.033

[20] Goode D Allen VM Barrow PA. Reduction of experimental Salmonella and Campylobacter contamination of chicken skin by application of lytic bacteriophages. Appl Environ Microbiol. (2003) 69:5032–6. doi: 10.1128/AEM.69.8.5032-5036.2003, 12902308 PMC169133

[21] Orquera S Gölz G Hertwig S. Control of *Campylobacter* spp. and *Yersinia enterocolitica* by virulent bacteriophages. J Mol Genet Med. (2012) 6:273–8. doi: 10.4172/1747-0862.1000049, 22872802 PMC3410378

[22] Rafiq MS Chen J Akmal M Ma D Asif A Wang J . Characterization and application of a novel Campylobacter phage CC_R7 as a biocontrol agent in chicken meat. Curr Res Food Sci. (2025) 11:101182. doi: 10.1016/j.crfs.2025.101182, 40896515 PMC12398825

[23] Fokas R Kalatzis PG Vantarakis A. Bacteriophages as food biocontrol agents: a one health framework for manufacturing quality, regulatory governance, and ethical stewardship—a narrative review. Viruses. (2026) 18:368. doi: 10.3390/v18030368, 41902276 PMC13030339

[24] Hue O Le Bouquin S Laisney M-J Allain V Lalande F Petetin I . Prevalence of and risk factors for Campylobacter spp. contamination of broiler chicken carcasses at the slaughterhouse. Food Microbiol. (2010) 27:992–9. doi: 10.1016/j.fm.2010.06.004, 20832676

[25] Althaus D Zweifel C Stephan R. Analysis of a poultry slaughter process: influence of process stages on the microbiological contamination of broiler carcasses. Ital J Food Safety. (2017) 6:7097. doi: 10.4081/ijfs.2017.7097, 29564242 PMC5850064

[26] European Food Safety Authority (EFSA), European Centre for Disease Prevention and Control (ECDC). The European Union one health 2024 zoonoses report. EFSA J. (2025) 23:e9759. doi: 10.2903/j.efsa.2025.9759 41377167 PMC12686834

[27] Noormohamed A Fakhr M. A higher prevalence rate of Campylobacter in retail beef livers compared to other beef and pork meat cuts. Int J Environ Res Public Health. (2013) 10:2058–68. doi: 10.3390/ijerph10052058, 23698698 PMC3709364

[28] Walker LJ Wallace RL Smith JJ Graham T Saputra T Symes S . Prevalence of *Campylobacter coli* and *Campylobacter jejuni* in retail chicken, beef, lamb, and pork products in three Australian states. J Food Prot. (2019) 82:2126–34. doi: 10.4315/0362-028X.JFP-19-146, 31729918

[29] Strachan NJC MacRae M Thomson A Rotariu O Ogden ID Forbes KJ. Source attribution, prevalence and enumeration of *Campylobacter* spp. from retail liver. Int J Food Microbiol. (2012) 153:234–6. doi: 10.1016/j.ijfoodmicro.2011.10.033, 22133565

[30] Regulation (EU) (2019) 2019/1243 of the European Parliament and of the Council of 20 June 2019 adapting a number of legal acts providing for the use of the regulatory procedure with scrutiny to articles 290 and 291 of the treaty on the functioning of the European Union (text with EEA relevance). Available online at: http://data.europa.eu/eli/reg/2019/1243/oj (Accessed May 31, 2026)

[31] Gao F Tu L Chen M Chen H Zhang X Zhuang Y . Erythromycin resistance of clinical *Campylobacter jejuni* and *Campylobacter coli* in Shanghai, China. Front Microbiol. (2023) 14:1145581. doi: 10.3389/fmicb.2023.1145581, 37260688 PMC10229067

[32] Abdraimova N Shitikov E Kornienko M. The role of genomics in advancing and standardising bacteriophage therapy. Antibiotics. (2026) 15:55. doi: 10.3390/antibiotics15010055, 41594091 PMC12838229

[33] Commission Implementing Regulation (EU) (2025) 2025/1390 of 15 July 2025 concerning the authorisation of a preparation of the bacteriophages PCM F/00069, PCM F/00070, PCM F/00071 and PCM F/00097 as a feed additive for poultry (holder of authorisation: Proteon Pharmaceuticals S.A.). Available online at: http://data.europa.eu/eli/reg_impl/2025/1390/oj (Accessed May 31, 2026)

[34] EFSA Panel on Biological Hazards (BIOHAZ), EFSA Panel on Contaminants in the Food Chain (CONTAM), EFSA Panel on Animal Health and Welfare (AHAW). Scientific opinion on the public health hazards to be covered by inspection of meat (poultry). EFSA J. (2012) 10:2741. doi: 10.2903/j.efsa.2012.2741

[35] Guyard-Nicodème M Payen C Larivière-Gauthier G Mompelat S Quesne S Anis N . Co-inoculation of broilers by Campylobacter and Salmonella: effect on colonization, cecal microbiota, and serum metabolome. Microbiol Spectrum. (2026) 14:e01102–25. doi: 10.1128/spectrum.01102-25, 41649264 PMC12955424

[36] Chang VP Mills EW Cutter CN. Reduction of Bacteria on pork carcasses associated with chilling method. J Food Prot. (2003) 66:1019–24. doi: 10.4315/0362-028X-66.6.1019, 12801003

[37] Choi D Ryu S Kong M. Phage-derived proteins: advancing food safety through biocontrol and detection of foodborne pathogens. Comp Rev Food Sci Food Safe. (2025) 24:e70124. doi: 10.1111/1541-4337.70124, 39898971 PMC11891642

[38] Gencay YE Jasinskytė D Robert C Semsey S Martínez V Petersen AØ . Engineered phage with antibacterial CRISPR–Cas selectively reduce *E. coli* burden in mice. Nat Biotechnol. (2024) 42:265–74. doi: 10.1038/s41587-023-01759-y, 37142704 PMC10869271

[39] Loh B Gondil VS Manohar P Khan FM Yang H Leptihn S. Encapsulation and delivery of therapeutic phages. Appl Environ Microbiol. (2021) 87:e01979–20. doi: 10.1128/AEM.01979-20, 33310718 PMC8090888

[40] Zamora PF Reidy TG Armbruster CR Sun M Van Tyne D Turner PE . Lytic bacteriophages induce the secretion of antiviral and proinflammatory cytokines from human respiratory epithelial cells. PLoS Biol. (2024) 22:e3002566. doi: 10.1371/journal.pbio.3002566, 38652717 PMC11037538

[41] Pang C Yin X Zhang G Liu S Zhou J Li J . Current progress and prospects of enzyme technologies in future foods. Syst Microbiol Biomanuf. (2021) 1:24–32. doi: 10.1007/s43393-020-00008-6

